# Gallbladder cancer with EGFR mutation and its response to GemOx with erlotinib: a case report and review of literature

**DOI:** 10.1186/s12957-020-01934-4

**Published:** 2020-07-04

**Authors:** Kishan Soni, Tarun Kumar, Manoj Pandey

**Affiliations:** Department of Surgical Oncology, Institute of Medical Sciences, Banaras Hindu University, Varanasi, 221005 India

**Keywords:** Gallbladder cancer, Tyrosine kinase inhibitors, Gemcitabine, Erlotinib, Chemotherapy

## Abstract

**Background:**

Gallbladder cancer (GBC) is the most common and aggressive extra hepatic biliary tree cancer (BTC) with dismal outcome. Complete surgical resection is the treatment of choice. Chemotherapy is used for palliation in advanced GBC where surgery is not possible, and the most commonly used agent is gemcitabine in combination with cisplatin or oxaliplatin or with capecitabine regimens. Complete remissions are hardly encountered in these cases; therefore, it is important to combine standard therapies with molecular targeting.

**Case presentation:**

A 60-year-old woman presented with pain in abdomen and loss of appetite for 1 month, and imaging showed locally advanced gallbladder carcinoma with liver metastasis. After biopsy confirmation, patient was initially started on gemcitabine and oxaliplatin combination followed by gene sequencing, which showed Tp53 (exon 7—c.713 G > A and exon 5—c.376-2A > G) and EGFR (exon 20—T790M) mutation, and erlotinib was added to chemotherapy, after 6 cycles of chemotherapy patient showed a 90% partial radiological response as per RECIST criteria.

**Conclusion:**

This case reports the possible efficacy of erlotinib in combination with gemcitabine and oxaliplatin in treating an EGFR*-*mutated GBC with liver metastasis. To our knowledge, this is the first article reporting the response to erlotinib combination therapy with this particular solitary mutation.

## Introduction

Gallbladder cancer (GBC) is the most common biliary tree cancer (BTC) with the short median survival, affecting more women than men. Higher incidence GBC has been reported from Chile, Japan, Poland, and northern India [1]. The disease is characterized by local invasion into common bile duct (CBD), liver, duodenum, and colon with or without vascular encasement, regional lymph node metastasis, and distant metastases commonly to liver and lung. Peritoneal carcinomatosis with omental and mesenteric deposits and ascitis is also commonly seen. Complete surgical resection is the most preferred treatment of choice in GBC cases. However, advanced unresectable cases require chemotherapy and have a poor prognosis. Combination chemotherapy with gemcitabine and cisplatin (GC) is the standard of care for advanced BTC [2]. Complete remission is hardly encountered in these cases with chemotherapy; therefore, it is important to combine standard treatment with molecular targeted drugs.

The search for molecular targets led to identification of epidermal growth factor receptor (EGFR) and vascular endothelial growth factor receptors (VEGF) in biliary cancers [3]. Other than p53, ErbB signaling (including EGFR, HER2, ERBB3, and ERBB4) is the most commonly mutated pathway, affecting up to 36.8% of the GBC [4]. The EGFR protein expression is seen in 54 to 65% of BTC [5] and is found to be associated with disease progression and poor prognosis [4–6]. Cell line studies have shown that by blocking HER1/EGFR tyrosine kinase signaling by addition of TKIs to gemcitabine, it decreases the growth of BTC and improves the anticancer effect of gemcitabine [7]. Erlotinib a first-generation EGFR TKI is currently approved for patients with non-small cell lung cancer and first line of treatment for metastatic or locally advanced pancreatic cancer in combination with gemcitabine [8].

Oxaliplatin differs in pharmacokinetics and dynamics from other platinum derivatives, like cisplatin and carboplatin, and has been used in combination with gemcitabine in clinical studies [9].

Gene sequencing has identified EGFR mutations in East Asians never smokers, and women having adenocarcinomas [10]. Retrospective studies show that 80% of patients with EGFR mutant non-small cell lung cancer (NSCLC) tumors treated with EGFR TKIs show radiographic and clinical responses with better progression free survival (PFS) and overall survival (OS) compared to wild type [11, 12]. Results of the studies show that not all tumors with activating EGFR mutations respond to EGFR inhibitors. Activation of EGFR also occurs with exon 20 inframe base pair insertions; however, this is also associated with de novo resistance to EGFR TKIs [13, 14].

Review of literature revealed a handful of studies that has evaluated the effect of erlotinib in EGFR-mutated GBC patients. A randomized phase III study showed that addition of erlotinib to gemcitabine and oxaliplatin enhances anti-tumor activity, which might become a treatment option for patients with cholangiocarcinoma [15]. Another phase II study showed that combining sorafenib and erlotinib does not improve response rate in BTC advocating patient selection based on molecular markers [3]. A case of stage IV gallbladder cancer with no EGFR mutation having long complete response to treatment with EGFR-TKI plus chemotherapy has been reported [16]. Therefore, addition of erlotinib to standard therapy may provide a more efficacious treatment for GBC patients with metastasis.

In the present case, next generation sequencing (NGS) was performed and analyzed in terms of mutations, amplifications, and over expression. The following is the first case report demonstrating the effect of erlotinib with gemcitabine-oxaliplatin regimen in a EGFR-mutated (exon 20 T790M) GBC patient with liver metastasis which showed a complete radiologial response to liver metastasis and 90% partial response in primary disease, with 11 months PFS.

## Case report

**Fig. 1:**
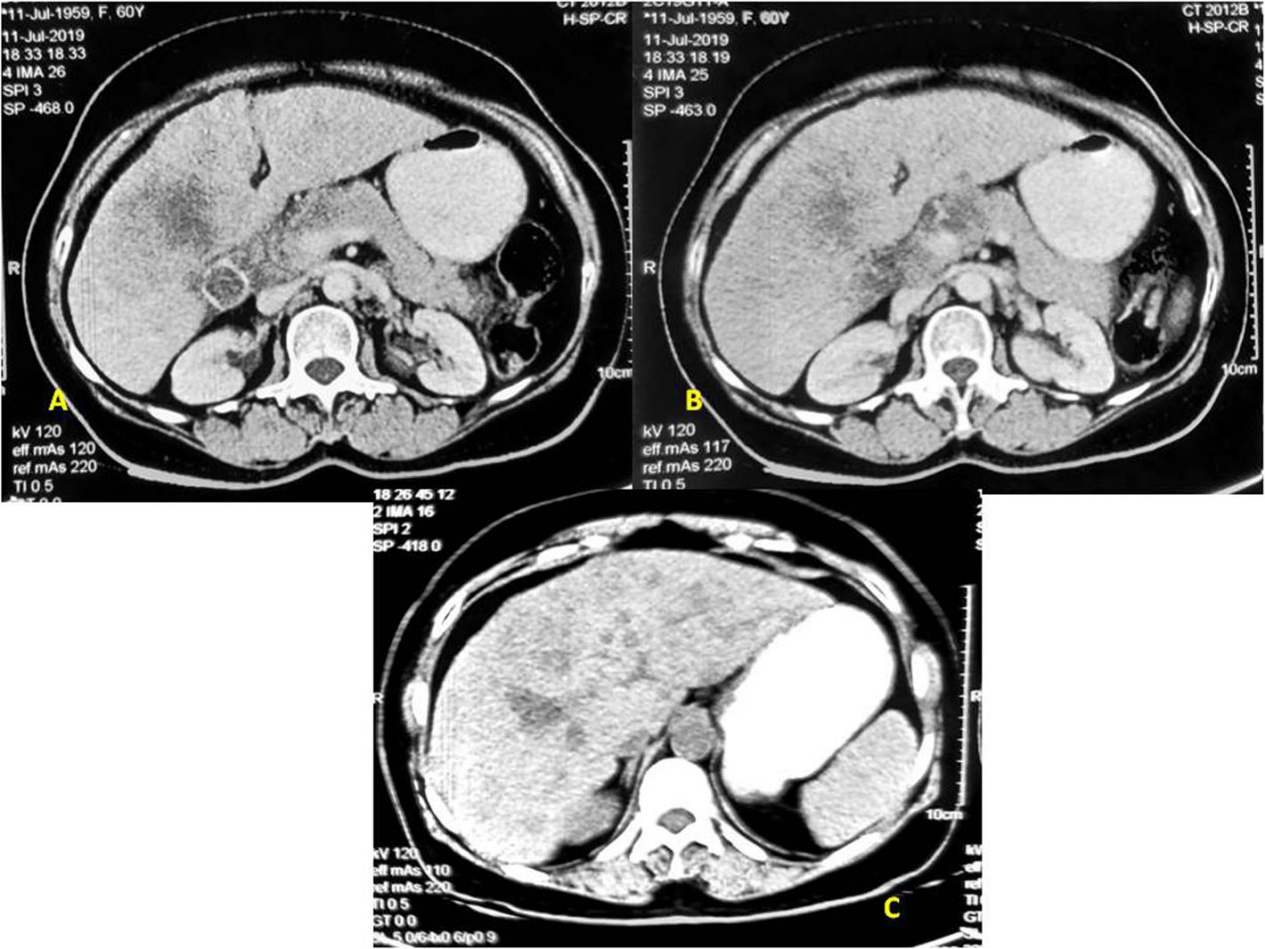
Pretreatment CT scan showing **a** mass in the fundus of gallbladder infiltrating IVB, V segment of liver with gallstones. **b** Liver metastasis with periportal and precaval lymphadenopathy. **c** Extensive liver metastasis

In July 2019, a 60-year-old female presented with chief complaint of abdominal pain and loss of appetite for 1 month in surgical oncology OPD of Sir Sundarlal Hospital, Institute of Medical Sciences, Banaras Hindu University (Varanasi-India). There was no history of co-morbidities like diabetes, hypertension, tuberculosis or asthma, and also did not have icterus. There was no past history of surgical treatment. Physical examination revealed mild right hypochondriac tenderness. Contrast enhanced computed tomography (CECT) revealed gallbladder (GB) distension, calculus, calcification of GB wall, irregular inhomogenously enhancing GB wall thickening, and multiple hypo-dense lesions in both lobes of the liver, multiple enlarged mesenteric and peri-pancreatic lymph nodes (Fig. 1a–c). Image-guided biopsy revealed adenocarcinoma of gallbladder. Routine complete blood count and serum biochemistry were within normal limits, whereas CA 19-9 level was 39.2 (upper limit of normal 35 IU/mL). Patient was advised for next generation sequencing (NGS) which came positive for Tp53 (exon 7—c.713 G > A and exon 5—c.376-2A > G) and EGFR (exon 20—T790M).

### Treatment regimen and results

**Fig. 2:**
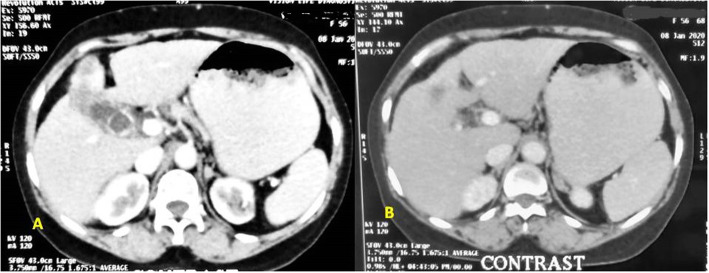
Post treatment scan showing **a** residual mass in the fundus of gallbladder with normal liver and **b** residual mass in segment V of gallbladder with normal liver and absence of nodes

Initially, patient was treated with two cycles of fixed rate gemcitabine 1 gm/m^2^ administered on day 1 and day 8 and oxaliplatin 100 mg/m^2^ intra venous on day 1 every 21 days. From 3rd cycle onwards after assessment of NGS report, a daily dose of erlotinib 150 mg was added to above mentioned treatment regime up to 6 cycles of chemotherapy which concluded in January 2020. After completion of chemotherapy, a CECT scan of abdomen was performed. It showed no evidence of disease in the liver having normal size, shape, outline, and CT attenuation (Fig. 2a), showing a complete radiological response as per RECIST criteria. GB showed multiple calculi in lumen, eccentric wall thickening in fundo-body region infiltrating segment IVB and V of liver (Fig. 2a–b) suggesting 90% response as per RECIST. CA19-9 at this juncture was 17.3. During the course of the disease, grade I neutropenia and grade I skin rashes were seen. Patient was shifted to maintenance therapy with capecitabine 1500 mg in divided doses for 14 days and erlotinib 150 mg per oral once daily. Patient is tolerating the treatment well and has not shown any further disease progression 11 months after initiation of treatment.

## Discussion

The above case displays the efficacy of combination of erlotinib with gemcitabine and oxaliplatin in treating a EGFR-mutated GBC with liver metastasis. From the past two decades, erlotinib has been gaining considerable attention in the field of cancer therapeutics. There is documentation of few intriguing studies, which are elementary to further research in this concern. In a randomized phase III study, Lee et al. compared 133 patients receiving only chemotherapy with 135 patients receiving chemotherapy in combination with erlotinib. The median PFS was 4 months versus 5.8 months. In patients with cholangiocarcinoma, the combination resulted in better PFS compared to GBC suggesting molecular differences between two cancer subtypes [15]. Mody et al. reported a complete remission by the fourth cycle of therapy in a case of metastatic, wild-type EGFR, gallbladder cancer infiltrating liver with lymph node metastasis treated with gemcitabine and erlotinib, and the treatment was continued for 12 cycles. They suggested that future studies of EGFR-TKI therapy plus chemotherapy in patients with BTCs should not be restricted only to those patients with EGFR mutations [16].

The “first-generation’ EGFR inhibitors, i.e., gefitinib and erlotinib, are reversible, ATP-competitive inhibitors that target exon 19 deletion and L858R mutant EGFR selectively and have been used in the treatment of EGFR-mutant NSCLC and pancreatic cancer, achieving up to a 72% response rate (RR) and nearly 10 months PFS [17–19]. A second exon 20, T790M mutation in EGFR, in patients already receiving first generation TKI, accounts for nearly half of the acquired resistance to first-generation TKI [20–23]. The proposed mechanism of resistance is steric hindrance imposed by the presence of a methionine residue preventing the binding of first-generation TKI to EGFR. However, further studies showed that the affinity of the mutant EGFR receptor to gefitinib is decreased but the binding is not completely inhibited [24, 25]. In 2013, a case of adenocarcinoma lung with EGFR exon 20 insertion A763_Y764insFQEA has been reported, which was responsive to erlotinib that was added to gemcitabine, docetaxel, and vinorelbine regimen.

Our case being GBC with T790M mutation in EGFR exon 20 showed a near complete response and prolonged PFS, with the use of first generation EGFR TKI erlotinib added to the gemcitabine-oxaliplatin regimen, which is a significant phenomenon. We cannot attribute the observed response solely to treatment with erlotinib, and erlotinib when added to gemcitabine has been shown to increase its efficacy and that could have resulted in response in this case. In BTC, a 13–15% EGFR mutation has been reported suggesting that only such patients be enrolled in clinical trials [26, 27]. Iyer et al. showed that patients with KRAS (G12V) mutation do not respond to treatment and should be excluded, but, the same was not found for KRAS (G13D) mutation [28]. There is still uncertainity over the exact mechanism and role of these mutations and response to EGFR TKI’s, further detailed studies, and clinical trials with large sample size and improvised patient selection are required to confirm such benefits of the treatment for GBC.

## Conclusions

The present case report demonstrates a complete response in liver metastasis and 90% partial response to primary disease in the presence of a tumor-associated EGFR mutation in patient with metastatic gallbladder cancer. Our findings highlight the clinical response in EGFR exon 20, T790M mutations with EGFR TKI erlotinib in combination with chemotherapy. Even though it cannot be concluded that this response is solely due to addition of erlotinib, nonetheless, it strengthens the need for future studies of EGFR-TKI therapy plus chemotherapy in patients with GBC in patients with EGFR mutations.

## Data Availability

Not applicable as all information and data is presented in manuscript.
